# Closing remarks: novel approaches to complex societal change and sustainability

**DOI:** 10.1007/s11625-018-0581-2

**Published:** 2018-05-29

**Authors:** Sander van der Leeuw

**Affiliations:** 1 Arizona State University, Tempe, USA; 2 Santa Fe Institute, Santa Fe, USA

**Keywords:** ICSS2017, Future earth, Sustainability

## Abstract

This paper summarizes some personal impressions of the 7th conference of the International Complex Systems Society, co-organized with “Future Earth”, held in Stockholm on August 24–26, 2017. The main point is that it is urgent and important to consider the sustainability conundrum as long-term, society-driven one, and to place societal dynamics at the core of how we, as a global society, came to this point, how ongoing dynamics are driving us towards a tipping point, and which role the Information and Communication Technology revolution plays in that process. A much wider involvement of the social sciences is essential. This also requires major changes in our thinking about sustainability—we need to develop an approach in which change is the natural state of affairs and societies attempt to impose stability on the dynamics involved. We need to focus on learning from the past, about the present, but above all for the future. And we need to shift from an entity-focused approach to a relational one, which pays more attention to contexts and networks. Other issues raised by such a shift in our thinking are about the role of science, the adoption of complex systems approaches and a few others that the paper points to.

## Introduction

This conference was for me a very pleasant surprise. I have been involved from very early on both in the history of ICSS, as one of its founding members, and in the emergence of Future Earth after the demise of IHDP, IGBP and other earlier Global Environmental Change research communities. At this conference, I observed early signs of a convergence among some of the very important changes in the Global Environmental Change community that both ICSS and Future Earth have been working towards for a number of years.

These changes concerned first of all a rejuvenation of the community, with the presence and input of many younger researchers and in particular also the active participation of many women, and second a much more diverse and sizable participation in the discussions on the part of scientists and scholars from the developing world.

But the changes I observed went much deeper. Some of the sessions presented successful attempts at co-design, showing ways to achieve intellectual fusion among scientists and scholars of different disciplines [e.g. KAN session “Mobilising multi-scalar energy sector transformations”], and between these and various representatives of stakeholder communities [e.g. session II: “Innovation and knowledge systems”].

Other sessions showed a growing acknowledgement that the dynamics that we are dealing with are in effect very complex, “hairy” ones that need to be approached by means of Complex (Adaptive) Systems thinking [e.g. KAN session on “Urban tinkering”].

To my mind one of the tangible changes in thinking also concerned the fact that both the natural and life sciences and the social sciences were (in my opinion) for the first time meeting each other on a more or less equal basis in both the research done and in the debates during the conference (for example the “Framing the conference” session).

In the following pages, I will comment on some of these things in more detail, but before I do so, I need to position myself and my intellectual outlook. I am an archaeologist and (medieval) historian who has, for a considerable period now, developed a Complex Adaptive Systems perspective on many sustainability-related challenges (van der Leeuw 1998).

The perspective that I bring to bear on the sustainability conundrum sees it as a current manifestation of developments that have occurred throughout history in many societies. Creative destruction has always been part of human history, and it is an inherent part of all human societal dynamics, whether we like that or not […….]. Humanity has gone through many such phases of creative destruction. Among the ones that are most commonly acknowledged I need only mention the demise of the Roman Empire, the Sassanian Empire, the Empire of the Moghuls, and numerous others. Tainter has first drawn attention to this in his (now famous) “*Collapse of Complex Societies*” (1988). Nothing new, therefore, except that this time it concerns the society in which we are living.

Part of the perspective that I bring to bear on these dynamics is a long-term optimism, coupled with a short-term pessimism. Humanity has thus far always found a solution for its most pressing problems *in the nick of time*, under pressure of circumstance. But in the short term, that is the next decades or century, I am more of a pessimist, as I think the major changes that need to occur will be difficult to implement and will entail major collateral damage.

## A long-term perspective on the sustainability conundrum

The current sustainability conundrum is part of a much longer history, and ignoring that biases our approach in unfortunate ways. Its main driver is the co-evolution of human cognition, societal organization and society’s engagement with the environment (Read and van der Leeuw 2015).

The most relevant phase of this development begins with the discovery and harnessing of fossil energy in the run-up to the Industrial Revolution. Fossil energy reduced the energetic cost of implementing inventions and it is, therefore, no surprise that with effect from the Industrial Revolution, innovation in our western societies hugely increased in frequency, and began spreading beyond the western world (Wrigley 2011). *That trend gave birth to the technology*-*dominated world of today and many of our sustainability challenges.*

Some details of that process are relevant here. In the 1830s–1840s, a major change occurred, initially in Great Britain, inverting the balance between society and economy (Polanyi 1944; Munck 2004; Graeber 2001; Frieden 2006). Whereas until that time, the economy in societies had generally been at the service of the society, from this period onward *society became in myriad ways dedicated to the well*-*being of the economy*, thereby laying the basis for the current market-based conception of society that is essentially focused on the acquisition of wealth.

A second change occurred after the Second World War, when a new global political order was created (Bretton Woods, United Nations, etc.) to deal with the tensions between capital and labor (Haass 2017). As part of that institutional reordering, growth in wealth and consumption became an essential part of our current societal dynamic, so that *consumerism thus became an essential feature of the modern developed world*, with all the attendant consequences for our Earth and its resources (Durning 1992).

The third major change that we have to take into consideration is the current Information Technology Revolution, It will have similarly fundamental, unpredictable consequences for our societies as it concerns the foundations of our human societal organization, which is shaped by the way we collectively process information. As a result, everything about human societies will change to the point of becoming unrecognizable (Friedman 2016; Brynjolfsson and McAfee 2011; Ito and Howe 2016), and that this well happen in the same time-frame as the impact of climate- and environmental change on our Earth. Beware!

## The real challenge is societal, not environmental

Indeed, the social science community now has reached a more or less equal position, vis-à-vis the natural and life sciences, in the domain of sustainability studies. As part of that development, sustainability is now viewed as a socio-environmental challenge and much research is dedicated to the immediate relationship of societal and environmental dynamics: so far, so good. But we must now go a step further and finally acknowledge that *the real sustainability challenge is societal, not environmental.* After all, societies define what they consider their environments, what they see as the main challenges in the environment, and what kind of solutions they can try and offer. If humanity is to re-equilibrate with the environment, that will have to come from societal changes, changes in mindset and societal structure. As Luhmann argued a while ago (1989): societies do not communicate with their environment—they communicate among their members about what they see as their environment, and they do so self-referentially.

This shift—*placing society at the center of the sustainability debate*—is the next, necessary quantum jump that we have to implement in our thinking.^1^ It, and only it, will stop us going round and round without getting anywhere. At the current conference, the participation of a younger generation, more women and more colleagues from the developing world has already shown some shifts towards fundamental criticism of the existing societal world order, including a stronger focus on transition dynamics.

This reflects a number of ‘heavy’ tendencies that are already transforming our societies, some of which have been mentioned in the conference:

- Consumerism is confronted with limited resources and the need to reduce pollution
- The “developed” world is being overtaken by cumulated debt and by wealth discrepancies, leading to potential financial and social instabilities.
- There is an active global megatrend towards producing more volume at lower prices, driving economies to the bottom, which is ultimately unsustainable
- Our current explosive population growth is environmentally and socially unsustainable

I conclude that the shift towards a new societal mindset will come in its own good time, willy-nilly, no matter what we do as scientists (or politicians, for that matter). Societies are forced to move in that direction by forces beyond their control! The best we can do as scientists and scholars is to begin to think seriously about the kinds of changes that the shift will entail, and how we might best contribute to anticipating and mitigating their impact on society.

As mentioned, the ICT revolution is adding a major, accelerating, dynamic in the mix: the loss of the distinction between signal and noise. Because anyone can now communicate with anyone in the world, be it one-on-one, one-on-many or many-on-many, anyone can now establish a community of people with a set of values according to their own taste. As a result, the alignment on certain basic values that has until now been responsible for a degree of societal coherence in our societies is being eroded away. The way in which that has emerged in recent politics is the idea of “alternative truths” propagated by government officials who no longer believe in the values of the majority of their societies. Ultimately, this trend is likely to inaugurate a phase of relative chaos in our societies before other sets of values can emerge.

## Change and stability

Much of our current, western, societies are based on an Aristotelian approach to the world around us, which assumes stability interrupted by phases of change (“punctured equilibria”, Eldredge and Gould 1972). Currently, the future seems to be catching up with us, or rather we seem to be eating our future (Flannery 2002), so we can no longer delay focusing on a society subject to permanent change. Some of this shift is already emerging in the “circular economy”, but this trend will of necessity overtake much of our interaction with the environment.

Hence, for the 21st century, we would do well to consider in more detail the perspective of Heraclitus of Ephesus (535 BC–475 BC): “Everything always changes, nothing remains the same”. Making that mental change has a number of important implications for our sciences.

For centuries, most of our studies have focused on the origins of the present. This is driven by the fact that to have a career in science, ever since the foundation of the Royal Society in 1664, one is required to prove every step of one’s argument. As it is impossible to prove anything for the future, scientific careers have predominantly focused on the relationship between present and past, elaborating the origins of a present that was assumed to be stable by invoking dynamics in the past.

In more recent years, a growing emphasis has emerged on the study of ways to understand and stimulate innovation and change, rather than understand stability (Fagerberg et al. 2013). But the existing paradigm is less than suitable for such studies—its focus on origins of existing phenomena rather than the emergence of novelty allowed us to improve our understanding of the conditions under which innovation happens, and its consequences, but did not really clarify the dynamics of invention themselves. Understanding these requires an approach that assumes permanent change; one that focuses on feed-*forward* and *anticipation* rather than feed-*back*! (Dearing et al. 2010; van der Leeuw et al. 2011)

Assuming that change is permanent and omnipresent, but interspersed with phases of stability, also shifts our perspective *on the role of innovation*. Rather than emphasize the study of the dynamics of change, once change is assumed one will be drawn to the study of the conditions for stability. Modern evolutionary theory in biology is a good example: it has clearly concluded that both stability and change are the result of a complex regulatory dynamic that can engender phases of both. Understanding such regulatory dynamics in the societal domain would be a major step forward (Laubichler and Renn 2015).

## Some difficulties in changing our thinking

As we come to introduce this novel approach, we are hampered by some of the ways to process information that we have acquired as part of the existing paradigm. It is characteristic of every paradigm that the thinking within a discipline or school of thought is limited by the questions and issues that have never been raised within it (Kuhn 1962).

In our case, the existing approach to sustainability has served to limit change. Much of our resilience and sustainability thinking is aimed at enabling our societies to continue on their current trajectories while reducing or limiting the negative impacts of the ways we do things: CO_2_ emissions, ocean acidification, pollution and waste dispersal, etc.

The approach that I think will inevitably emerge will be one in which our thinking must permanently adapt to the changes that are occurring, rather than try to limit or control them. That is the true essence of the Complex Adaptive System thinking that accepts the ontological uncertainty of our future (Mitchell 2011; Lane and Maxfield 2005). It requires that we emotionally accept the importance of ambiguity and uncertainty in a society that has for centuries tried to focus on certainty and dis-ambiguation. We must embrace change and design for it! Our worst enemy in this domain is our fear of fear itself.

Another aspect of that transition is a necessary move from an intellectual approach focused on defining and arguing in terms of entities to an approach that includes an emphasis on relationships. Here, we can learn, in the West, from the ways people in East Asia cognize phenomena. When confronted with a complex image that represents both a subject and its context, westerners seem to focus on the subject and retain that better than the context, whereas in East Asia, the context is better retained and the subject less precisely (Nisbett 2003; Acar et al. 2011). Whatever the reason (and one can think of the complexities of learning a Chinese or Japanese script against the relative simplicity of distinguishing a western alphabet), the difference is fundamental, as considering the context is inevitably relational, whereas the subject is an entity.

Hence, there are different pattern recognition techniques involved, and we would do well, in a world in which change is assumed, to acquire ways to deal with the contextual nature of all kinds of change and reinforce our skills in understanding complex patterns.

## Tinkering and complex systems

Jacob’s (1977) paper “*Evolution and tinkering*”, which was referred to by several of the participants (Day 2, session “Tinkering in an urban context”), is fundamentally a plea for adopting a Complex Adaptive Systems approach. It assumes that the world is so complex that we will never be able to control its dynamics, so that many of the outcomes of any human interaction with the environment are unpredictable and best considered as “unintended consequences” of those interactions. Change is unforeseeable, ad hoc, and rather than trying to construct or control outcomes, humans should adapt to, and live with, the changes that their (and other dynamics’) interactions are triggering. Human impact, in Jacob’s vision, is best described as ‘tinkering” (“bricolage”), and has at best a very limited impact on the overall dynamics. Jacob builds on an argument that was first launched by Monod (1970), who argued that complex processes auto-regulate themselves for considerable periods of time, creating an appearance of stability because the dynamics involved are so strong that deviation seems impossible, whatever humans try to do. But at certain times, these strong dynamics reach “tipping points” at which they do no longer drive the system in any particular direction and, at such moments, human action can indeed impact on the direction processes take. Most of you will recognize this as very similar to the perspective of Holling and the Resilience Alliance (and Research Center) (Gunderson and Holling 2002).

I am emphasizing that because this approach forces us to admit a degree of humility in what science can actually do to change the large-scale complex socio-environmental dynamics that the Earth system is undergoing in the Anthropocene. “Tinkering” is taking the old, recycling, modifying it, reusing it with a (partially) different function. It implies a continued use of experimentation and learning from experiments, including according value to experimental failure. It relies more upon human and social capital, and uses them better, while reducing our reliance on material capital. Although in this context it may seem novel, it is not: most European, Asian and African cities were built that way, and many people love them for it.

## Power to … power over … power to

The role of power was touched upon a number of times in this conference but was not really central to any of the discussions. I nevertheless want to devote a few lines to it, as we can no longer avoid according it its natural role in our discussions. My long-term perspective on these matters sees current dynamics as the late impact of the Euro-American colonial system that began in the 16th century and spread worldwide in the following years until the 2nd World War and its aftermath. This globalization for centuries drove a transition from local “power to” (achieve results) to global “power over” in the hands of a few nations and their ruling classes. In the process, local, homegrown sustainability was generally replaced by global, imposed unsustainability.

In the 20th century, this process changed in form, from military to economic, but did not change in essence. 20th century globalization forcibly aligned the many different cultures with their wide-open stable social value systems into a single global value system ubiquitously based on the lowest common denominator of all these cultural value systems: ‘wealth’ and ‘money’.

The reduction in dimensionality triggered two dynamics: first, a competition to become the ‘best’ in the only remaining dimension along which individuals and nations now compared themselves (wealth). This is driving the growing wealth gap that is currently becoming a threat to societal coherence. Second, growing popular resistance to current trends, as people no longer feel they share a group or cultural identity, while at the same time feeling devalued as individuals because they have lost their personal identity.

For social wellbeing worldwide, it is essential that people rediscover their personal and group identities, and this requires that societies rediscover the full dimensionality of their value systems. As part of that process, individuals and groups need to assume their societal responsibilities by being politically active. That, and only that, will both bring societal stability and resilience, and “free up people’s animal spirits” of entrepreneurship and optimism.

## It’s a major battle!

The entrenched ‘power over’ system located in the multinational corporations and the governments that either actively support or condone them, as well as subconsciously in most of our minds, is extremely powerful. The transformation it will—without doubt—undergo will not be accepted smoothly, although an increasing number of businesses and governments are beginning to see the need for change. In short, the battle between the two worldviews will be rude.

In many instances, governments and societies have in the last fifty years lost control and functionality to corporations. In the West, maybe the most powerful sign of that were the consecutive “big bangs” in New York and London in the early 1980s, when governments in the US and UK gave up control over the financial world.

As part of globalization, many communities have been destroyed as part of the migration towards towns, and the fact that governments took over healthcare and care for the old, thus undermining one of the major functions of local communities worldwide: mutual assistance. If we are to regain our ‘power over’ as citizens, we need to rebuild communities, networks, local institutions and local value systems. Those are the new challenges of co-design and co-implementation.

But maybe most importantly, our own thinking has for so long been part of that system that we have difficulty distinguishing in our own thoughts and actions how it impacts all we think and do. One of the major and tenacious dimensions of that phenomenon is our assumption that our global demography, health, technology, etc. are on a linear growth trajectory. That assumption is firmly rooted in the “idea of progress” that western cultures have adopted centuries ago!

## The role of scientists

As I have repeatedly said, major transitions are on the way and the world will change whether scientists contribute to these changes or not. Unfortunately, our scientific enterprise is functionally suboptimal. It has reached a state of stagnant tolerance. The social sciences missed their window of opportunity to become well organized, contrary to the natural and life sciences who grabbed that opportunity in the last half of the 20th century. The community of social scientists is fragmented and introverted. Career structures are functioning as straitjackets, and so is the funding structure for science. Indeed, science has lost control over its own destiny, and has become an instrument of control in the hands of industry and governments.

The current loss of trust in science is in part due to the fact that it overpromised miracles in the 1940s–1970s, while at the same time some of the unintended consequences of its advances were highly negative in the eyes of society. But there are other, more fundamental reasons, which we ignored to our detriment. Paramount among those is the fact that the general public judges the results of science contextually. And in the modern world that means that many scientists are seen as part of the techno-industrial complex that pays for their work; however, independent and controversial their results may be. In many places that is an affiliation that those in society who have no scientific training, but developed different ways to explain the world to themselves, distrust (Wynne 1992a, b) …

## What can we do as scientists?

I do not think scientists of any kind can successfully try to change the world or the transformational trajectory it is on. That is a dangerous illusion. Scientists can do two kinds of things. First, they can ‘tinker’ in the margins of the major societal dynamics, and second (and maybe more usefully) they can try and alert our societies to the kinds of changes that are coming, so that people can begin to prepare themselves for these changes. That is the significance of “studying the back loop” (Gunderson and Holling 2002).

Part of the disconnect between scientists and the wider civil society is the fact that scientists are perceived as arrogant, while they are not aware that they are so perceived. They have been trained to strongly believe in the superiority, values and results of a particular way of thinking that does not resonate with non-scientists, and they are often not capable of (or inclined to) relativizing the contribution of that approach. Their great strength is in rationally reducing the dimensionality of complex phenomena to the point that clarity is gained, but in the process they often discard so many dimensions that their scientific thinking to an extent loses the relation to the reality it attempts to ‘explain’. Gregory Bateson’s son asks him (in the 1972 *“Steps to an Ecology of Mind*”): “Why do things always end up in a muddle?” Well, reality is so complex, and our cognition is so limited that it will always come across as a muddle! As scientists, we need to let go of the idea that we can do more than tinker at the margins.

But that should not discourage us in the practice of our (scientific) professions because alerting our societies to the kinds of changes that are potentially coming is in itself a very, very valuable contribution as it helps society prepare for dealing with them. Science can usefully accompany societal change, but should shed the idea that it can control it.

We can help such a transition by making our science more transparent, admitting both its strengths and its weaknesses, being explicit about our methods and their strengths and limitations, and communicating these. Under the impact of Mertonian philosophy (Merton 1942) we have set ourselves apart, arguing for an ‘objective’ and ‘even-handed’ way to interact with the rest of society. But we are no different from other citizens in society, except that we have been trained in a particular way of thinking that is as relevant to the dynamics of society as any other. Modesty about our role is the way forward!

## Bringing in outsiders

Awareness of the more modest role scientists should be playing in society entails also makes it easier to accept the need to bring in outsiders. Not only is the relatively small worldwide community of scientists insufficiently strong to engage in a major way in the forces that will shape our futures, but it is too narrowly focused to explore more than a very small part of the total possibility space of our future. Our societies need to have the fullest panoply of ideas and capabilities to experiment with, as is brought home powerfully in Ito and Howe in their recent book “Whiplash” (2016).

The contribution of outsiders is also important because they actually live with the consequences of their decisions, whereas scientists often point out the uncertainties, but then go home to devote themselves to other questions. Living in a world of self-generated risks teaches a degree of modesty about one’s potential impact on events. They have a much more holistic and high-dimensional approach to pattern recognition in the muddle that is the real world and are, therefore, likely to ground us scientists and scholars. That is a necessary precondition for a successful role of scientists and scholars in the society of the future.

## Conclusion

Summarizing my impressions of the conference in the current context of sustainability research, and in particular referring to the efforts of Future Earth to bring the science community out of its isolation into the wider world, I would point in the following directions:

- The conference testified to a serious effort at enlarging global participation in the discussions. I noticed a rejuvenation of the community, the active participation of many women, and a much more diverse and sizable participation on the part of scientists and scholars from the developing world. That had the very positive effect that the nature of the discussions themselves changed, bringing important societal issues to the fore that had until now only been discussed in the background, among a limited subset of sustainability scientists in the humanities and social sciences. This should in my opinion be a major driver of Future Earth efforts.
- Both the natural and life sciences and the social sciences were (in my opinion) for the first time meeting each other on a more or less equal basis in both the research done and in the debates during the conference. In part due to the influence of scholars from the developing world, there was an increased acknowledgment that many sustainability challenges are actually societal ones rather than environmental ones. However, the conference did not set the final step of accepting that the sustainability challenge needs to be seen as fundamentally the result of unanticipated consequences of societal dynamics. Societies define what they consider their environments, the challenges they observe in them, the solutions they propose, and the measures they take to achieve them. In furthering the acceptance of that basic truth Future Earth could play a very useful role.
- Another of Future Earth’s major goals is the development of co-design, showing ways to achieve intellectual fusion not only among scientists and scholars of different disciplines, but also between them and various representatives of stakeholder communities. This very important goal is a more difficult one to achieve. The differences between these worlds with respect to their goals, approaches and, more generally, ways of thinking are very, very substantive. Scientists and scholars tend to answer questions and then find new ones to answer. Most stakeholders in the wider community, on the other hand, focus on finding solutions rather than answer questions. This difference leads to important differences in the structure of research, and these are difficult to bridge. Much more focused work on this bridging effort is necessary, and calls for involving more specialists in that domain.
- A couple of the sessions showed a growing acknowledgement that the dynamics that we are dealing with are in effect very complex, “hairy” ones, and that the usual reductionist scientific approach is not helping us identify the dynamics involved. They need to be approached by means of Complex (Adaptive) Systems thinking. Although that approach has spread quite substantially in the physical and life sciences, the need for it is not yet very widely acknowledged in the social domain, which is often dealing with the most complex dynamics. Here again, Future Earth could contribute, for example by developing a KAN that is not looking at the symptoms of the current challenges, but digging deeper, across themes and disciplines, into some of the fundamental epistemological challenges of our current approaches.

Tempe, Arizona, September 12, 2017.

## Footnotes

1 It is remarkable that a Google search does not yield among the most cited references any that specifically makes this point (dd. March 22, 2018).
